# The Benefits of a Comprehensive Cardiac Rehabilitation Program for Patients with Acute Coronary Syndrome: A Follow-Up Study

**DOI:** 10.3390/jpm13101516

**Published:** 2023-10-21

**Authors:** Laura Maria Craciun, Florina Buleu, Ana Maria Pah, Marius Badalica-Petrescu, Olivia Bodea, Dana Emilia Man, Oana Catalina Cosor, Stela Iurciuc, Simona Dragan, Maria Rada

**Affiliations:** 1 Department of Cardiology, “Victor Babes” University of Medicine and Pharmacy, E. Murgu Square no. 2, 300041 Timisoara, Romania; dr.lauracraciun@gmail.com (L.M.C.); ana11p@yahoo.com (A.M.P.); marius_badalica@yahoo.com (M.B.-P.); olivia_maria88@yahoo.com (O.B.); danaemilia@yahoo.com (D.E.M.); oanacatalinaiancu@yahoo.com (O.C.C.); stela_iurciuc@yahoo.com (S.I.); simona.dragan@umft.ro (S.D.); radamariam@gmail.com (M.R.); 2 Research Center of the Timisoara Institute of Cardiovascular Diseases, “Victor Babes” University of Medicine and Pharmacy, 300041 Timisoara, Romania

**Keywords:** acute coronary syndrome, secondary prevention, comprehensive cardiac rehabilitation program, guideline-directed medical therapy

## Abstract

Background and objectives: Secondary prevention after acute coronary syndrome (ACS) is essential to reduce cardiovascular mortality and hospital readmission, ensuring patients return to normal with an improved quality of life. Thus, we investigate the benefits of a comprehensive cardiac rehabilitation (CR) program on lifestyle, risk factors and adherence to guideline-directed medical therapy (GDMT) in patients after ACS and myocardial revascularization through coronary artery by-pass grafting (CABG) or percutaneous coronary intervention (PCI). Methods: This is a prospective, longitudinal study in consecutive post-CABG or PCI patients after ACS that participated or not in a comprehensive CR. Cardiovascular risk factors, quality of life and adherence to GDMT were analyzed in terms of assessing the benefit of 12 months of comprehensive CR on reaching guidelines secondary prevention targets. Results: At the inclusion in comprehensive CR of all patients (*n* = 480), 85% had hypertension; 86% had elevated total cholesterol values; 69% were characterized by metabolic syndrome; 43% were obese; 31% were active smokers and 29% had type 2 diabetes mellitus. Only 26.66% (*n* = 128) followed the entire program for 12 months. No statistically significant differences in the prescription of GDMT at hospital discharge after myocardial revascularization between the CR (+) group (*n* = 128) versus CR (−) group (*n* = 352) (*p* > 0.05) were observed. After 12 moths, a significant adherence to GDMT in the CR (+) group vs. CR (−) group was recorded, as follows: antiplatelet agents (100% versus 96%, *p* = 0.001), beta-blockers (99% versus 92%, *p* = 0.02), ACE inhibitors/ARAB (89% versus 79%, *p* = 0.04), lipid-lowering drugs (100% versus 89%, *p* = 0.001). In total, 82% of the CR (+) patients had a significantly higher adherence at GDMT (82% versus 64%, *p* = 0.001). At 12 moths, the CR (+) group was characterized by significantly lower values than at the inclusion but some values still increased: systolic blood pressure (139.25 + 19.20 mmHg (*p* < 0.03)), total cholesterol (171.07 + 48.59 mg/dL (*p* = 0.0001)) and LDL-cholesterol (102.83 + 41.30 mg/dL (*p* = 0.009)). At the same time, the analysis of psychosocial factors using the HAD questionnaire revealed a statistically significant improvement in anxiety and depression scores: HAD-A score (9.1 ± 3.7 at T0 vs. 7.1 ± 4.2 at T1, *p* = 0.001) and HAD-D score (7.7 ± 3.19 at T0 vs. 6.4 ± 4.3 at T1, *p* = 0.003). A multivariable analysis, following GDMT, showed the actual value or information and training of patients regarding optimal cardiovascular risk factor control was independently associated with lower values of systolic blood pressure (R^2^ = 0.48), diastolic (R^2^ = 0.38), serum glucose (R^2^ = 0.48), glycated hemoglobin (R^2^ = 0.50), total cholesterol (R^2^ = 0.31), LDL-cholesterol (R^2^ = 0.30), HDL-cholesterol (R^2^ = 0.19) and serum triglycerides (R^2^ = 0.20). Conclusion: The twelve-month participation of post-ACS patients in comprehensive CR resulted in excellent post-revascularization management, as well as good adherence to guideline-directed medical therapy, provided further confirmation of the benefit of secondary prevention. Despite high adherence to drug treatments, targets for blood pressure, total cholesterol and LDL-cholesterol are inadequately achieved. Therefore, in the era of personalized medicine, patients with ACS should benefit from specific, comprehensive cardiovascular recovery programs that contain physiotherapists, psychologists, nutritionists and an experienced cardiologist in cardiovascular rehabilitation.

## 1. Introduction

Cardiovascular rehabilitation was first introduced in the 1960s only for low-risk patients who survived an acute myocardial infarction [1]. Evidence from randomized clinical trials over the past 3–4 decades supports contemporary clinical guidelines, which now recommend routine referral for cardiovascular rehabilitation, including for patients with heart failure, after acute coronary syndrome and coronary revascularization (percutaneous coronary intervention or coronary artery bypass surgery) [2,3,4,5].

Furthermore, especially through a comprehensive modification of risk factors, secondary prevention has brought evidence of the beneficial effects of a cardiovascular rehabilitation program [6]. Patients with acute coronary syndrome should be referred for a cardiovascular rehabilitation program as early as possible immediately after hospitalization. Each week of delay in starting the program requires an additional month of exercise to achieve the same level of health benefits [7,8].

Decreasing morbidity and mortality, increasing quality of life and reducing recurrence are the main objectives of secondary prevention after acute coronary syndrome. To achieve them, secondary prevention in the available guidelines for the clinical management of patients with cardiovascular diseases [2,3,4,5], the following are uniformly recommend: (1) the optimal use of cardio-protective drug therapies (antiplatelet agents, beta-blockers, angiotensin-converting enzyme (ACE) inhibitors/angiotensin II receptor blockers (ARA), lipid-lowering drugs), (2) lifestyle interventions with the optimal control of cardiovascular risk factors (blood pressure (BP), lipid profile and blood sugar values), psychosocial factors, smoking cessation, increased physical activity, maintaining a healthy body mass index (BMI) and nutrition [9,10]. Thus, the medical team carrying out the comprehensive cardiac rehabilitation program should include a physiotherapist, a nurse and an electroradiology technician, in addition to doctors. Cooperation with a psychologist and a dietitian is also important [5]. 

Studies that included patients after ACS confirmed the benefits of the cardiac rehabilitation program. Data showed that total mortality decreased by 20–25%, including that from cardiac causes [11]. At the same time, a 13% reduction in the risk of subsequent cardiac interventions was indicated [12]. Although clinical trials have proven CR’s effectiveness, further research needs to strengthen the evidence base for comprehensive cardiac rehabilitation in patients after acute coronary syndrome [13]. 

Therefore, the primary objective of this study was to investigate the benefit of 12 months of comprehensive CR on reaching the guidelines’ secondary prevention targets in patients with ACS who followed a comprehensive CR compared with those who did not. Second, this study aimed to examine the benefit of CR on cardiovascular risk factors, quality of life, psychological well-being and adherence to guideline-directed medical therapy.

## 2. Materials and Methods

### 2.1. Study Design and the Selection of Participants

This study was conducted in the Cardiovascular Prevention and Rehabilitation Clinic of the Institute of Cardiovascular Diseases Timișoara, Romania, and enrolled 480 consecutively hospitalized patients with acute coronary artery disease who showed an indication for undertaking a comprehensive cardiac rehabilitation program. All patients underwent diagnostic coronary angiography, followed by revascularization procedures, documented in individual case reports. Revascularization procedures were a percutaneous transluminal coronary angioplasty or a coronary artery bypass graft. The patients were no younger than 18 years and no older than 80 years. 

**Figure 1:**
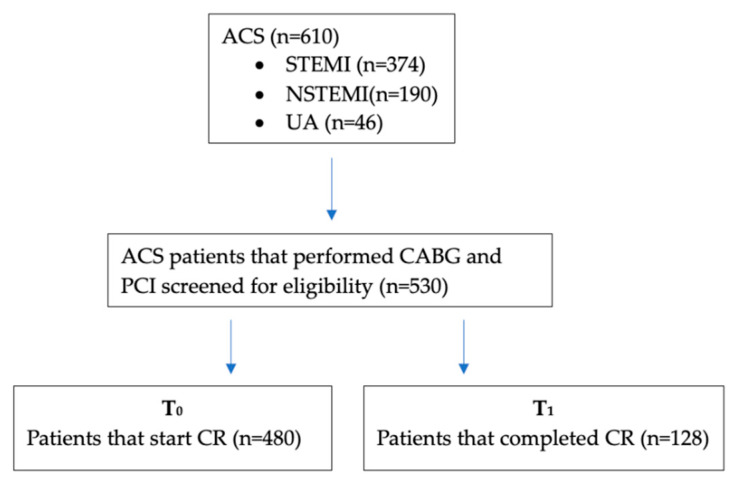
Patients with acute heart failure, degenerative valvular diseases, congenital heart diseases, cardiomyopathies of other causes than coronary artery disease, inflammatory diseases, infections or known neoplasms were excluded from the study group.

The initial sample included 610 cardiac patients after ACS, of whom 61.31% (*n* = 374) had ST-elevation myocardial infarction, 31.14% (*n* = 190) had non-ST-elevation myocardial infarction, and 46 patients presented with unstable angina. In total, 530 patients who underwent PCI or CABG were chosen to participate in the study; 480 of these patients agreed to start the comprehensive cardiovascular recovery program, but only 128 patients completed it (CR+). Age over 80 years (*n* = 2), a start period of the rehabilitation program greater than 1 month after ACS (*n* = 23) or death during the follow-up period (*n* = 12), as well as missing values for all relevant variables, were applied as criteria that led to the exclusion of 104 cases (Figure 1).

### 2.2. Clinical and Biochemical Evaluation

Demographic information, anthropometric parameters, personal medical history, cardiovascular risk factors and medications as well as laboratory values were collected at baseline and after completing the CR program. All patients also performed an assessment of psychological stress. 

The clinical evaluation included the measurement of systolic (SBP) and diastolic blood pressures (DBP) and body mass index. Blood pressure (BP) was determined according to the European Guidelines on cardiovascular disease prevention in clinical practice [5]. Body weight—W (kg)—was determined using a mechanical scale. Height—H (m)—was determined using a metal talimeter (Fazzini, Milan, Italy). The body mass index—BMI (kg/m^2^)—was calculated according to the following formula: BMI = weight (kg) ÷ height^2^ (m^2^). Abdominal circumference—AC (cm)—was determined using a metallic centimeter. Abdominal obesity was defined as abdominal circumference >94 cm in men and >80 cm in women [14]. For the determination of triglycerides, total cholesterol, LDL and HDL-cholesterol, photometric methods (Dimension RXL-MAX, Dade Behring, Erlangen, Germany) were used. For fasting blood glucose, the enzymatic method with hexokinase (HK) was applied, using Siemens reagents on a Dimension RXL-MAX, Dade Behring device, Erlangen, Germany. 

Psychological stress was quantified using the Hospital Anxiety and Depression Scale (HADS). The HADS [15] is composed of 14 items and contains two subscales, one for anxiety and another one for depression. Each item is quantified on a scale of 4 points on the Likert scale, from 0 (no symptoms) to 3 (maximum symptom level). The maximum score for each subscale is 21; scores 0–7 on each subscale are considered normal, while scores above 11 signify a considerable psychological morbidity, either anxiety (HAD-A) or depression (HAD-D). Scores 8–10 indicate a borderline status. Scores were considered if at least 5 responses were given for each subscale. Missing answers in patients who completed only 5 or 6 items were replaced based on the sum of the filled items multiplied by 7/5 and 7/6, respectively.

The questionnaire method was also used—it was based on different types of questionnaires addressed to the patients at the time of the interview (T_1_). All standardized questionnaires including a questionnaire regarding the management of cardiovascular risk factors (smoking, diet and body weight, arterial hypertension, hypercholesterolemia and diabetes); a questionnaire regarding awareness of cardiovascular risk factors and guideline recommendations and a questionnaire regarding the addressing of lifestyle change recommendations, respectively, complied with these recommendations. 

### 2.3. Intervention and Comprehensive Cardiac Rehabilitation Program

The comprehensive cardiac rehabilitation program started no earlier than 1 week and no later than 1 month from the time of acute coronary syndrome. The program included a multidisciplinary team which consisted of physiotherapists, psychologists, nutritionists and an experienced cardiologist in cardiovascular rehabilitation. The program included a bi-weekly 2 h session and continued for 12 months (T_1_). Each session included one or more group therapies based on cardiovascular risk factor education, physical therapy, dietary suggestions, aerobic exercise and anxiety and depression management. Exercise sessions lasted 30 min (exercise intensity was individually prescribed based on a target heart rate < 85% of theoretical threshold) and included warm-up and cool-down exercises.

Subsequently, the data of patients (*n* = 128) who completed the comprehensive cardiac rehabilitation program were analyzed for reaching the targets.

The targets of the secondary prevention program followed the recommendations of the guidelines of the European Society of Cardiology in force [5]:

- Smoking cessation;
- Systolic blood pressure  <  130 mmHg, if tolerated;
- LDL-cholesterol  <  55 mg/dL (<1.4 mmol/L);
- HbA1c  <  7% (53 mmol/L) for patients with diabetes mellitus;
- BMI  <  25 kg/m^2^;
- Physical activity of moderate intensity  ≥  150 min per week or vigorous intensity  ≥  75 min per week, or an equivalent by combining the two during the week;
- Daily use of lipid-lowering drugs/statins;
- Daily use of dual antiplatelet therapy;
- Mental health support in selected cases.

### 2.4. Data Analysis

Statistical analysis was performed using the SPSS software (SPSS 22.0 Inc., Chicago, IL, USA). Variables were expressed as means ± standard deviation or percentage results. Comparisons between groups were performed using the two-tailed Student *t*-test or ANOVA test as appropriate. Correlation coefficients were determined using linear regression analysis and statistical significance was determined with the Fisher test. Multivariable analyses were performed using stepwise linear regression or stepwise logistic regression as appropriate. A *p*-value < 0.05 was considered to be statistically significant. 

## 3. Results

### 3.1. Analysis of Baseline Data (T_0_) for All Patients (n = 480) 

From the total number of consecutive patients who met this study’s inclusion criteria (*n* = 480), 68.1% of them were revascularized using the interventional method PCI and 31.9% using the surgical method CABG. We noticed a male preponderance (74% men versus 26% women) of the analyzed group, with an average age of 61.7 + 9.66 years. The mean values for SBP were 147.32 ± 21.88 mmHg, for DBP 84.32 ± 25.74 mmHg, for MAP 105.32 ± 15.02 mmHg, for pulse pressure 63.0 ± 18.95 mmHg and for FBG 133.91 ± 56.59 mg/dL. Regarding the lipidic profile, the following mean values were recorded at baseline for all patients: 208.24 ± 51.75 mg/dL for TC, 125.62 ± 34.70 mg/dL for LDL-c, 39.52 ± 9.40 mg/dL for HDL-c and 183.95 ± 90.72 mg/dL for TG. After the assessment of anxiety and depression using HADS, we observed that the HAD-A mean values were 9.1 ± 4.52 and the HAD-D mean values were 7.5 ± 3.44 (Table 1).

Analyzing the main characteristics of all patients included in the studied group, we observed significantly higher values in females (*n* = 125) versus males (*n* = 355) for the following variables: age (*p* = 0.0004), body mass index (*p* = 0.004), systolic blood pressure (*p* = 0.009), pulse pressure (*p* = 0.01), total cholesterol (*p* = 0.0006), LDL-cholesterol (*p* = 0.008) and serum triglycerides (*p* = 0.02). None of these values fall within the hemodynamic and metabolic profile recommended by the European Guidelines for the secondary prevention of atherothrombotic cardiovascular disease, as can be seen in Table 2.

All patients analyzed (*n* = 480) at baseline were characterized by an increased cardiovascular risk profile at the time of ACS: 85% had hypertension; 86% had elevated total cholesterol values; 69% were characterized by metabolic syndrome; 43% were obese; 31% were active smokers; 29% had type 2 diabetes mellitus.

Regarding the prevalence of non-modifiable risk factors, 46% of patients had a family history of premature cardiovascular disease and 66% were over 55 years old for men and 65 years old for women.

**Figure 2:**
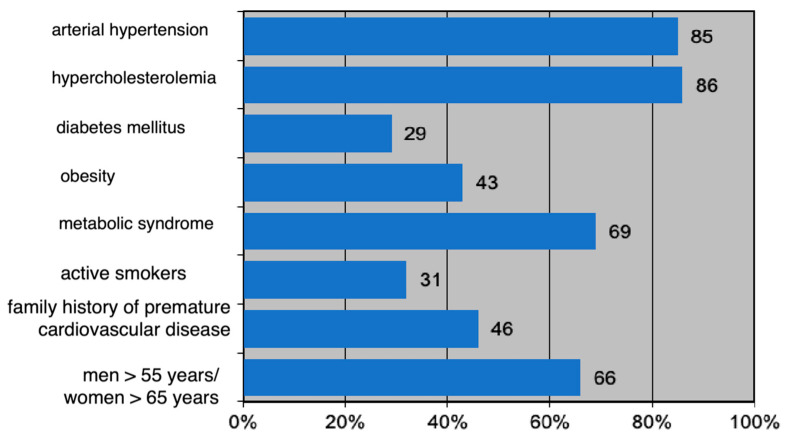
Epidemiological profile of the studied group (*n* = 480) expressed as percentages.

The epidemiological profile of the entire group (*n* = 480) is detailed in Figure 2.

### 3.2. Analysis of Data for Patients Included in the CR Program

All patients received the recommendation for the inclusion in the comprehensive cardiac rehabilitation program according to gender and type of intervention. Only 26.66% (*n* = 128) followed the entire program for 12 months. 

Comparing the variables analyzed in the group included in the comprehensive cardiac rehabilitation program (*n* = 128) versus the group that did not completed this program (*n* = 352) at the time T_0_ (inclusion in the program), we did not register statistically significant differences (Table 3).

Comparing the variables analyzed in the patient group that completed the CR program (*n* = 128) at T_0_ versus T_1_, the analysis revealed a significant decrease between the two moments for systolic blood pressure (146.64 + 25.65 mmHg versus 139.25 ± 19.20 mmHg, *p* = 0.03), total cholesterol (200.0 + 52.04 mg/dL versus 171.07 + 48.59 mg/dL, *p* = 0.0001), LDL-cholesterol (117.39 + 31.60 mg/dL versus 102.83 + 41.30 mg/dL, *p* = 0.009), HbA1c (6.3 ± 0.81% vs. 5.8 ± 0.65%, *p* = 0.004), HAD-A score (9.1 ± 3.7 at T_0_ vs. 7.1 ± 4.2 at T_1_, *p* = 0.001) and HAD-D score (7.7 ± 3.19 vs. 6.4 ± 4.3, *p* = 0.003).

We did not find any significant statistical differences (*p* < 0.05) between the two moments for the rest of the analyzed variables (body mass index, diastolic blood pressure, HDL-cholesterol, serum triglycerides and blood sugar). The results are presented in Table 4.

### 3.3. Analysis of Secondary Prevention Guideline-Directed Medical Therapy Adherence

**Figure 3:**
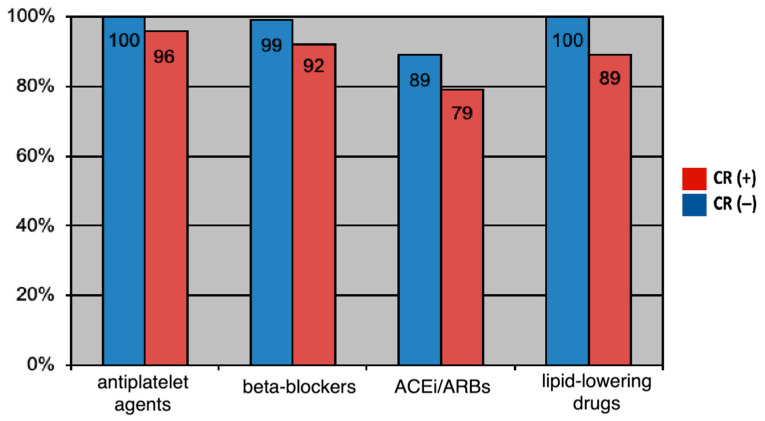
Secondary prevention guideline-directed medical therapy prescription at 12 moths after myocardial revascularization.

Although there were no statistically significant differences in the prescription of the guideline-directed medical therapy secondary prevention at hospital discharge after myocardial revascularization between the CR (+) group (*n* = 128) and the CR (−) group (*n* = 352) (*p* > 0.05), after 12 moths, we recorded a significant adherence to GDMT in coronary patients who participated in the comprehensive cardiac rehabilitation program versus non-participants as follows: antiplatelet agents (100% versus 96%, *p* = 0.001), beta-blockers (99% versus 92%, *p* = 0.02), angiotensin-converting enzyme inhibitors/angiotensin II receptor blocker (89% versus 79%, *p* = 0.04) and lipid-lowering drugs (100% versus 89%, *p* = 0.001). The results are detailed in Figure 3.

**Figure 4:**
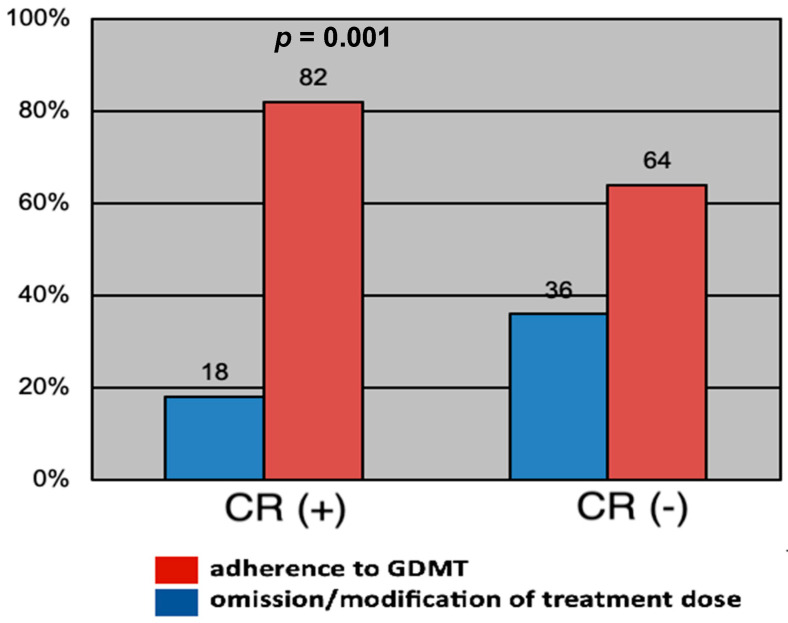
Adherence to guideline-directed medical therapy depending on participation or not in the comprehensive cardiac rehabilitation program.

Patients included in the comprehensive cardiac rehabilitation program showed a significantly higher adherence to guideline-directed medical therapy compared to the rest of the patients (82% versus 64%, *p* = 0.001), as can be seen in Figure 4. 

### 3.4. Analysis of the Proportion of Target Achievements

Comparing the proportion of those reaching their targets in the group included in the comprehensive cardiac rehabilitation program (*n* = 128) at T_0_ and T_1_, respectively, the analysis revealed a significant increase in reaching the target values for blood pressure (*p* = 0.00001), fasting blood (*p* = 0.001) and lipid profile parameters, and total cholesterol (*p* = 0.00001), LDL-cholesterol (*p* = 0.000007), HDL-cholesterol (*p* = 0.01), serum triglycerides (*p* = 0.0007) at T_1_ time (Table 5).

Comparing the proportion of reaching the targets in the group included in the comprehensive cardiac rehabilitation program (*n* = 128) versus the group that did not participate in this program (*n* = 352), the analysis revealed a significant increase in reaching the target values in the subgroup of coronary patients who benefited from the measures of a comprehensive secondary prevention within the program, for blood pressure (*p* = 0.0001), lipid profile parameters: total cholesterol (*p* = 0.00009), LDL-cholesterol (0.007), HDL-cholesterol (*p* = 0.002) and serum triglycerides (*p* = 0.002). We did not record significant differences between the two groups analyzed regarding reaching the values recommended by the guide for the anthropometric (body mass index) and glycemic (fasting blood glucose) parameters (Table 5).

### 3.5. The Benefit of the Comprehensive Cardiac Rehabilitation Program in Secondary Prevention

In order to quantify the benefit and the insufficient implementation of complex secondary prevention measures, we applied linear multivariate regression models for parametric variables, respectively, and logistics for categorical variables. After adjusting for statistically insignificant variables (*p* > 0.05), the following components of the comprehensive cardiac rehabilitation program remained in the final model and independently contributed to the improvement and achievement of targets, respectively (Table 6).

## 4. Discussion

The present study refers to a particular group of subjects, patients with ACS and myocardial revascularization, who present, according to the latest Guidelines for the Prevention of Cardiovascular Diseases of the European Society of Cardiology, a very high cardiovascular risk [5].

Starting from the abundant evidence from observational epidemiological studies that indicates a direct relationship between the increased prevalence of traditional risk factors and the increased rate of acute cardiovascular events, we can say that patients with ACS are a priority for cardiovascular secondary prevention [16]. 

Comparing the results of the EUROASPIRE (European Action on Secondary Prevention by Intervention to Reduce Events) studies, a unique picture was provided in Europe regarding the prevalence of cardiovascular risk factors post myocardial revascularization and the need to intensify lifestyle change measures in this category of patients was emphasized [17]. 

Similarly, the TASPIC-CRO I-V (treatment and secondary prevention of ischemic coronary events in Croatia) studies revealed a progressive increase in the prevalence of diabetes, hypertension and obesity among interventional or surgical coronary revascularization in 35 medical centers in Croatia [18]. Comparing the results from TASPIC-CRO V versus EUROASPIRE II, a higher prevalence of cardiovascular risk factors is observed in the first study: arterial hypertension (69% versus 54%), diabetes (30% versus 22%), hypercholesterolemia (65% versus 59%), respectively smoking (34% versus 21%) [17,18].

The same increased prevalence and inadequate control of cardiovascular risk factors among surgically/interventionally revascularized coronary patients (in particular, the control rate of blood pressure), was also recorded in the Survey of Risk Factors in Coronary Heart Disease (SURF CHD) II study, developed in collaboration with the European Society of Cardiology and the European Association of Preventive Cardiology. The SURF CHD II study was recently conducted in countries from Europe, Asia, the Middle East, North Africa and South America to assess the recording and monitoring of cardiovascular risk factors in patients with established coronary artery disease and secondary prevention indication, and to evaluate the effectiveness of the implementation of and adherence to clinical guidelines in daily practice [19]. 

The patients analyzed by us were characterized at the time of inclusion in the study by an increased prevalence of arterial hypertension (85%); moreover, for the values of the marked risk hemodynamic profile (systolic blood pressure, diastolic blood pressure, pulse pressure or mean arterial pressure), the recommended target value was not reached. Also, the lipid profile of the patients included in this study was proatherogenic; the achievement of the target values recommended by current guidelines for all lipid parameters was below 50%. Along with the increased cardiometabolic and hemodynamic risk profile, an additional risk was found in the increased prevalence of active smoking post myocardial revascularization after ACS (31%).

The increased prevalence of arterial hypertension (85%) observed in this study is all the more worrying and higher as the reported results from a similar study conducted almost 10 years ago. Data were analyzed from the ISACS-TC (International Survey of Acute Coronary Syndromes in Transitional Countries) registry and included 2286 retrospective Romanian patients hospitalized with a diagnosis of ACS in 23 Romanian hospitals. It was revealed that 1450 patients were hypertensive, thus meaning a prevalence of arterial hypertension of 63.4% among ACS patients [20]. This value is higher than that reported for the general population of Romanians (40.4%) [21]. Since elevated blood pressure values remain among the most influential cardiovascular risk factors, an extensive attempt to provide the optimal antihypertensive medical approach should also be considered an important goal in secondary prevention. Recently, the identification of the underlying genetic pathways and mechanisms responsible for the induction of hypertension, as well as the gender-based individualization of antihypertensive therapy, has provided evidence of the improved rates of accurate blood pressure control [22].

In this study, in order to quantify the benefit of pharmacological and non-pharmacological measures in reducing cardiovascular risk, 12 months after inclusion in the comprehensive cardiac rehabilitation program the general characteristics of these patients were analyzed. We found that the CR (+) group was characterized at the time of the reassessment (T_1_) by significantly lower values than at the time of T_0_ (inclusion) but still increased values of systolic blood pressure (139.25 + 19.20 mmHg (*p* < 0.03)), total cholesterol (171.07 + 48.59 mg/dL (*p* = 0.0001)) and LDL-cholesterol (102.83 + 41.30 mg/dL (*p* = 0.009)). At the same time, the analysis of psychosocial factors using the HAD questionnaire revealed a statistically significant improvement in anxiety and depression scores: HAD-A score (9.1 ± 3.7 at T_0_ vs. 7.1 ± 4.2 at T_1_, *p* = 0.001) and HAD-D score (7.7 ± 3.19 at T_0_ vs. 6.4 ± 4.3 at T_1_, *p* = 0.003).

Our results are consistent with the data from the literature.

The GENDER (Genetic Determinants of Restenosis) study, which included over 3000 coronary patients undergoing PCI for an acute coronary syndrome, showed the same inefficiency of secondary prophylaxis measures; 86% of patients with metabolic syndrome and 60% of those without metabolic syndrome had elevated blood pressure values 9 months after the myocardial revascularization procedure [23]. Similarly, a study that analyzed secondary prevention led by Urbinati et al., which included 11,706 patients with acute myocardial infarction, found that, at six months after the acute event, despite a high adherence to drug treatments, blood pressure, LDL-cholesterol and diabetic targets recommended by guidelines were inadequately achieved [24]. Also, nearly half of patients ≥ 60 years of age with ACS did not reach their LDL-cholesterol goal after 3 months of secondary prevention, and suboptimal LDL-cholesterol control was more common in women [25].

We also demonstrated, in this study, the importance of psychoeducational rehabilitation from the comprehensive program in the secondary prevention of patients after ACS (by a statistically significant improvement in anxiety and depression scores for CR (+) patients), which, according to a review that included 3090 participants from 11 studies, was associated with the improvement of new non-cardiovascular events, quality of life, most cardiovascular risk factors, physical behavior and mental health outcomes such as depression, anxiety and distress, along with illness perception and cognitions [26].

The beneficial trend of our research was represented by the drastic reduction in the percentage of smokers in the CR (+) group (from 30% at T_0_ to 13% at T_1_), this fact being the consequence of the recommendations made in this regard along with the awareness of the risks represented by continued smoking.

A systematic review that included a total of 39 studies with 11,228 patients included in a program of secondary prevention after ACS showed that the cumulative smoking cessation rate was 45.0%. Most smokers with ACS continued to smoke after discharge. Patients who participated in cardiac rehabilitation programs quit smoking to a greater extent, while people with depression and those with chronic lung diseases quit smoking less. These patient categories should be targeted for intensive interventions to quit smoking [27]. 

Despite the global consensus on the management of ACS, the implementation of strategies to improve adherence to guideline-directed medical therapy remains sub-optimal, particularly in developing countries [28]. When the discharge recommendations of 1072 patients with ACS were analyzed regarding the justifiable and unjustifiable omissions of mandatory drugs according to the prevention guidelines, the first month’s audit revealed unreasonable omissions of antiplatelet agents, statins, ACE-I/ARAB and beta-blockers in 1%, 0%, 14% and 11%, respectively, which reduced to 0% for all pharmacological agents by the end of the 11th month of the secondary prevention program. This improvement remained unchanged until the end of the 12th month after ACS [29]. Recently, numerous other drug attempts to reduce cardiac outcomes after ACS have been carried out [30,31,32,33,34,35], but not all of them have proven their effectiveness, which proves the recommendation of the guidelines in secondary prevention all the more.

Although, in the present study, we observed a significant increase in the prescription of guideline-directed medical therapy in revascularized coronary patients, 12 months after myocardial revascularization, 9% of patients did not receive the optimal maximum medication. The patients included in the comprehensive cardiac rehabilitation program benefited from medication optimization through periodic evaluations and showed a significantly higher compliance with the pharmacological treatment than the rest of the patients (*p* < 0.05) (Figure 3), thus showing that adherence for 1 year to a comprehensive CR program provides the confirmation of the benefit of such a secondary prevention program. 

Our results are similar to those of the Italian Cardiac Rehabilitation and Secondary Prevention after Cardiac Revascularization Survey (ICAROS), which also showed that participation in CR resulted in excellent treatment after revascularization, as well as good lifestyle and medication adherence at 1 year, providing further confirmation of the benefit of the secondary prevention program [36].

Although this study proved the benefits of the comprehensive cardiovascular rehabilitation program in patients after ACS, there is still room for improvement and, in a call to action, the identified gaps in secondary prevention must be addressed. It is clear that any new ideas and/or methods are warranted if we want to improve overall secondary prevention programs in these patients. There are many barriers to secondary prevention. We list just a few, focusing on those encountered in our study: the willingness of patients to participate in such programs, inequalities regarding the use and availability of secondary prevention medication and lack of adherence to secondary preventive pharmacological treatment.

Furthermore, the present study demonstrates a substantial deficit in the importance given to risk factors by both medical staff and patients. A possible explanation could be found in the misperception of interventional cardiologists and cardiovascular surgeons that their role consists only in treating the acute event without giving additional importance to the assessment and management of existing cardiovascular risk factors, thus creating insufficient implementation of comprehensive cardiac rehabilitation programs.

A reason for the low CR participation and completion rate among our patients, we believe, is the shorter length of hospital stay for patients with interventional coronary revascularization (24–48 h) compared to patients with surgical coronary revascularization (4–7 days). There is insufficient time to educate them on how to reduce cardiovascular risk or the importance of follow-up and inclusion in a comprehensive cardiovascular rehabilitation program.

At the same time, in order to quantify the benefit and avoid the insufficient implementation of complex secondary prevention measures, we applied linear multivariate regression models for parametric variables and logistics for categorical variables (Table 6). We noticed overall that knowing the recommended and actual value for guidelines targets independently contributed to the improvement and achievement of these, especially as insufficient understanding of cardiovascular disease among affected people is also prevalent, and patients tend to have an optimistic bias towards their own risk [37]. 

Since the hospitalization of patients with acute coronary syndrome and myocardial revascularization is short, they end up benefiting from evidence-based therapy provided by ambulatory cardiac rehabilitation programs or by initiating and continuing their rehabilitation in specialized centers. In a systematic review of 63 trials in which patients were randomized to receive either a comprehensive cardiac rehabilitation program or conventional care, a 12 months assessment after the initiation of secondary prevention showed a reduction in cardiovascular mortality (RR 0.74; 95% CI [0.64–0.86]) and hospital re-admissions (RR 0.82; 95% CI [0.70–0.96]) in the CR cohort. Interestingly, the observed benefits were consistent between intervention type (comprehensive cardiac rehabilitation program versus exercise-based rehabilitation) and rehabilitation care setting (at a specialized center, at home, or a combination of both) [38]. When the impact of in-hospital cardiac rehabilitation (IH-CR) on medication adherence, as well as on other cardiovascular outcomes, following an ACS in a group of 13,540 patients was analyzed, only 1101 patients (8%) onset a cardiac rehabilitation program within 30 days of discharge following the acute event [39], suggesting the need to identify and correct the barriers to CR participation for this higher risk group of patients. In our study, the comprehensive cardiac rehabilitation program was initiated and continued in a specialized local hospital and conducted by a cardiologist. This could be another added value of the current study. 

### Study Limitations

A weakness of this study is that the results were derived from a single regional specialized cardiac rehabilitation hospital from Romania, so they cannot be generalized to other contexts due to the differences in healthcare policies. Moreover, patients over 80 years old were not included because presence of such age-related changes in other organ systems may influence prognosis and adherence to guideline-directed medical therapy [40,41,42,43,44]. Another, limitation is that the analyzed batch is characterized by a lack of homogeneity in terms of gender distribution, with the proportion of male subjects (74%) and those undergoing interventional revascularization (68%) being clearly higher. 

It is also acknowledged that the accuracy of all self-reported information could not be fully verified. Patients overestimate their compliance with lifestyle change recommendations; when the evaluation is completed using the questionnaire method, as in this context, the results of clinical data expressed and the relationship with cardiovascular risk can be greatly underestimated. However, we performed quality control on a random sample of 20% of the questionnaire data and this indicated that the variation between self-reported and recorded information was small.

## 5. Conclusions

In the present study, 12 months after ACS and inclusion in a comprehensive CR, despite high adherence to guideline-directed medical therapy, achievement of target BP, total cholesterol, and LDL-cholesterol guidelines targets goals were inadequately achieved. Participation in a comprehensive CR after discharge and referral to a cardiac rehabilitation specialized hospital was associated with better adherence to a healthy lifestyle. This study also showed that a comprehensive cardiovascular rehabilitation program, which combined both information and education of patients with a view to awareness of cardiovascular risk factors, pharmacological and non-pharmacological measures of secondary prevention, as well as organized programs of physical training and adequate psycho-social management, had a beneficial approach and along with the long-term follow-up of these patients. Therefore, in the era of personalized medicine, patients with ACS should benefit from specific, comprehensive cardiovascular recovery programs that contain physiotherapists, psychologists, nutritionists and an experienced cardiologist in cardiovascular rehabilitation.

## Figures and Tables

**Table 1:** Characteristics of the entire analyzed group (*n* = 480) at baseline.

Variable	Mean ± Standard Deviation (SD)
Age (years)	61.7 ± 9.66
BMI (kg/m^2^)	29.99 ± 4.79
SBP (mmHg)	147.32 ± 21.88
DBP (mmHg)	84.32 ± 25.74
MAP (mmHg)	105.32 ± 15.02
PP (mmHg)	63.0 ± 18.95
TC (mg/dL)	208.24 ± 51.75
LDL-c (mg/dL)	125.62 ± 34.70
HDL-c (mg/dL)	39.52 ± 9.40
TG (mg/dL)	183.95 ± 90.72
FBG (mg/dL)	133.91 ± 56.59
HbA1c (%)	6.1 ± 0.91
HAD-A	9.1 ± 4.52
HAD-D	7.5 ± 3.44

BMI, body mass index; SBP, systolic blood pressure; DBP, diastolic blood pressure; MAP, mean arterial pressure; PP, pulse pressure; TC, total cholesterol; HDL-c, high-density lipoprotein cholesterol; LDL-c, low-density lipoprotein cholesterol; TG, triglycerides; FBG, fasting blood glucose; HbA1c %, glycated hemoglobin; HAD-A, Hospital Anxiety and Depression Scale for Anxiety; HAD-D, Hospital Anxiety and Depression Scale for Depression. Values were expressed as mean ± standard deviation (SD).

**Table 2:** Characteristics of the analyzed group according to gender and revascularization method (*n* = 480).

Variable	Males *n* = 355	Females *n* = 125	*p*	CABG *n* = 153	PCI *n* = 327	*p*
Age (years)	60.77 ± 9.67	64.33 ± 9.16	**0.0004 ^s^**	63.28 ± 8.60	60.95 ± 10.04	**0.013 ^s^**
BMI (kg/m^2^)	29.62 ± 4.57	31.04 ± 5.25	**0.004 ^s^**	29.90 ± 4.84	30.03 ± 4.77	0.7
SBP (mmHg)	145.52 ± 24.36	152.44 ± 28.81	**0.009 ^s^**	146.94 ± 25.95	147.50 ± 25.68	0.8
DBP (mmHg)	83.77 ± 10.96	85.88 ± 12.00	0.07	83.78 ± 10.98	84.57 ± 11.41	0.4
MAP (mmHg)	104.35 ± 14.33	108.06 ± 16.59	**0.01 ^s^**	104.83 ± 14.89	105.55 ± 15.10	0.6
PP (mmHg)	61.75 ± 18.06	66.56 ± 20.95	**0.01 ^s^**	63.15 ± 19.33	62.93 ± 18.79	0.9
TC (mg/dL)	203.42 ± 50.81	221.92 ± 52.18	**0.0006 ^s^**	205.98 ± 52.26	209.29 ± 51.56	0.5
LDL-c (mg/dL)	123.14 ± 34.76	132.68 ± 33.67	**0.008 ^s^**	123.86 ± 36.20	126.45 ± 34.0	0.4
HDL-c (mg/dL)	39.09 ± 9.36	40.72 ± 9.43	0.09	40.21 ± 9.94	39.19 ± 9.13	0.2
TG (mg/dL)	178.49 ± 89.91	199.45 ± 91.57	**0.02 ^s^**	190.37 ± 92.92	180.95 ± 89.65	0.2
FBG (mg/dL)	130.98 + 53.33	142.26 ± 64.46	0.05	132.98 ± 51.69	134.35 + 58.81	0.8
HbA1c (%)	6.1 ± 0.79	6.1 ± 0.73	0.088	6.1 ± 0.92	6.1 ± 0.75	0.7
HAD-A	9.0 ± 3.9	9.2 ± 2.97	0.090	10.2 ± 2.9	9.1 ± 3.24	0.2
HAD-D	7.6 ± 3.21	7.7 ± 3.34	0.383	8.8 ± 3.7	8.7 ± 3.34	0.383

BMI, body mass index; SBP, systolic blood pressure; DBP, diastolic blood pressure; MAP, mean arterial pressure; PP, pulse pressure; TC, total cholesterol; HDL-c, high-density lipoprotein cholesterol; LDL-c, low-density lipoprotein cholesterol; TG, triglycerides; FBG, fasting blood glucose; HbA1c %, glycated hemoglobin; HAD-A, Hospital Anxiety and Depression Scale for Anxiety; HAD-D, Hospital Anxiety and Depression Scale for Depression. Values were expressed as mean ± standard deviation (SD); s—signification.

**Table 3:** Characteristics of the analyzed group according to participation (*n* = 128) or not in the comprehensive cardiac rehabilitation program (*n* = 352) (T_0_).

Variable	CR (+) T_0_ *n* = 128	CR (−) T_0_ *n* = 352	*p*
Age (years)	61.43 ± 9.72	61.49 ± 9.68	0.9
BMI (kg/m^2^)	29.65 ± 4.58	29.75 ± 4.69	0.8
SBP (mmHg)	146.64 ± 25.65	147.35 ± 26.02	0.8
DBP (mmHg)	83.97 ± 11.12	84.25 ± 11.30	0.8
MAP (mmHg)	104.86 ± 14.78	105.28 ± 15.18	0.8
PP (mmHg)	62.67 ± 19.35	63.09 ± 19.00	0.8
TC (mg/dL)	200.0 ± 52.04	209.24 ± 51.97	0.1
LDL-c (mg/dL)	117.39 ± 31.60	127.55 ± 35.66	0.9
HDL-c (mg/dL)	39.11 ± 10.05	39.84 ± 9.12	0.5
TG (mg/dL)	177.54 ± 82.99	181.94 ± 90.57	0.6
FBG (mg/dL)	125.81 ± 46.57	134.05 ± 56.20	0.2
HbA1c (%)	6.3 ± 0.81	6.2 ± 0.94	0.4
HAD-A	9.1 ± 3.7	9.2 ± 2.85	0.3
HAD-D	7.7 ± 3.19	7.7 ± 3.24	0.4

BMI, body mass index; SBP, systolic blood pressure; DBP, diastolic blood pressure; MAP, mean arterial pressure; PP, pulse pressure; TC, total cholesterol; HDL-c, high-density lipoprotein cholesterol; LDL-c, low-density lipoprotein cholesterol; TG, triglycerides; FBG, fasting blood glucose; HbA1c %, glycated hemoglobin; HAD-A, Hospital Anxiety and Depression Scale for Anxiety; HAD-D, Hospital Anxiety and Depression Scale for Depression. Values were expressed as mean ± standard deviation (SD).

**Table 4:** Characteristics of the group included in the comprehensive cardiac rehabilitation program (T_0_ versus T_1_).

Variable	CR (+) T_0_ *n* = 128	CR (+) T_1_ *n* = 128	*p*
Age (years)	61.43 ± 9.72	62.67 ± 9.94	0.4
BMI (kg/m^2^)	29.65 ± 4.58	29.59 ± 4.27	0.9
SBP (mmHg)	**146.64 ± 25.65**	**139.25 ± 19.20**	**0.03 ^s^**
DBP (mmHg)	83.97 ± 11.12	81.26 ± 11.76	0.1
MAP (mmHg)	104.86 ± 14.78	101.19 ± 12.96	0.08
PP (mmHg)	62.67 ± 19.35	58.10 ± 14.26	0.07
TC (mg/dL)	**200.0 ± 52.04**	**171.07 ± 48.59**	**0.0001 ^s^**
LDL-c (mg/dL)	**117.39 ± 31.60**	**102.83 ± 41.30**	**0.009 ^s^**
HDL-c (mg/dL)	39.11 ± 10.05	40.53 ± 9.25	0.3
TG (mg/dL)	177.54 ± 82.99	152.88 ± 93.99	0.06
FBG (mg/dL)	125.81 ± 46.57	119.85 ± 44.72	0.3
HbA1c (%)	**6.3 ± 0.81**	**5.8 ± 0.65**	**0.004 ^s^**
HAD-A	**9.1 ± 3.7**	**7.1 ± 4.2**	**0.001 ^s^**
HAD-D	** 7.7 ± 3.19 **	**6.4** ** ± 4.3 **	**0.003 ^s^**

BMI, body mass index; SBP, systolic blood pressure; DBP, diastolic blood pressure; MAP, mean arterial pressure; PP, pulse pressure; TC, total cholesterol; HDL-c, high-density lipoprotein cholesterol; LDL-c, low-density lipoprotein cholesterol; TG, triglycerides; FBG, fasting blood glucose; HbA1c %, glycated hemoglobin; HAD-A, Hospital Anxiety and Depression Scale for Anxiety; HAD-D, Hospital Anxiety and Depression Scale for Depression. Values were expressed as mean ± standard deviation (SD); s—signification.

**Table 5:** Target achievement in the comprehensive cardiac rehabilitation program group (T_0_ versus T_1_) vs. non-participants (T_1_ for both).

Parameters	CR (+) T_0_ (%)	CR (+)T_1_ (%)	*p*	OR	(IC)	CR (−) T_1_ (%)	*p*	OR	(IC)
BMI < 25 kg/m^2^	12.5	11.36	0.8	1.11	0.41–3.03	15.6	0.4	1.34	0.63–2.94
TC < 155 mg/dL	15.9	54.54	** 0.00001 **	0.16	0.07–0.34	34.3	**0.00009**	0.40	0.24–0.65
LDL < 55 mg/dL	7.9	39.77	** 0.000007 **	0.13	0.05–0.34	27.2	** 0.007 **	0.52	0.31–0.87
HDL-c > 40 mg/dL (men); >50 mg/dL (women)	37.5	55.68	** 0.01 **	0.48	0.25–0.91	44.3	** 0.002 **	0.59	0.36–0.97
BP < 130/80 mmHg	9	40.9	** 0.00001 **	0.14	0.06–0.36	22.7	** 0.0001 **	0.39	0.23–0.66
TG < 150 mg/dL	42	71.59	** 0.00007 **	0.29	0.15–0.56	31.9	** 0.002 **	0.56	0.32–0.95
FBG < 110 mg/dL	42	67	** 0.001 **	0.36	0.18–0.69	51	0.05	0.63	0.37–1.05
Active smoker	30	13	**0.005**	0.32	0.17–0.58	38.63	** 0.00001 **	0.17	0.06–0.09

BMI, body mass index; BP, blood pressure; TC, total cholesterol; HDL-c, high-density lipoprotein cholesterol; LDL-c, low-density lipoprotein cholesterol; TG, triglycerides; FBG, fasting blood glucose.

**Table 6:** Components that contributed independently to the improvement and achievement of the comprehensive CR targets.

**Body Mass Index (R^2^ = 0.52)**	***p***	**β**
Following the dietary weight loss recommendation	0.04	0.8
Following weight loss through physical exercise recommendation	<0.0001	1.95
Knowing the ideal body weight	<0.0001	1.35
** Abdominal circumference (R^2^ = 0.41) **		
Following the dietary weight loss recommendation	0.04	2.18
Following weight loss through physical exercise recommendation	0.002	2.5
Knowing the ideal body weight	0.007	2.22
Informing the patient that he/she is overweight or obese	<0.001	6.13
Maintaining the proposed weight for more than 6 months	0.004	2.9
Advising and educating patients with diabetes in relation to glycemic control	0.01	2.17
** Systolic blood pressure (R^2^ = 0.48) **		
Following the comprehensive cardiac rehabilitation program	0.006	5.8
Following the recommendation to reduce salt consumption	0.0005	7.53
Knowing the recommended value for blood pressure	<0.0001	22.73
Knowing the actual value for blood pressure	0.04	3.38
Information and training of hypertensive patients regarding optimal BP control	<0.0001	9.3
** Diastolic blood pressure (R^2^ = 0.38) **		
The recommendation to increase the consumption of fish oil	0.003	3.25
Knowing the actual value for blood pressure	<0.0001	8.63
Information and training of hypertensive patients regarding optimal BP control	<0.0001	3.99
** Total cholesterol (R^2^ = 0.31) **		
The recommendation to reduce fat consumption	0.04	14.7
Compliance with the recommendation for weight loss through diet	0.01	11.23
Knowing the actual value of total cholesterol	<0.0001	17.37
Maintaining the proposed weight for more than 6 months	0.01	12.1
Informing and training dyslipidemic patients regarding the optimal control of lipid parameters	<0.0001	28
Following treatment with statin	<0.0001	46.5
** LDL-cholesterol (R^2^ = 0.30) **		
The recommendation to reduce fat consumption	0.007	16.65
Following weight loss recommendations by increasing physical activity levels	0.02	9.94
Knowing the actual value of total cholesterol	0.005	12.32
Maintaining the proposed weight for more than 6 months	0.04	8.28
Following treatment with statin	<0.0001	40.59
** HDL-cholesterol (R^2^ = 0.19) **		
The recommendation to increase the consumption of fish oil	0.009	2.26
The recommendation to reduce excessive alcohol consumption	0.01	3.03
Following weight loss recommendations by increasing physical activity levels	0.04	1.73
Following treatment with statin	<0.0001	3.64
** Triglycerides (R^2^ = 0.20) **		
The recommendation to increase the consumption of fish oil	0.01	23.55
Recommendation for weight loss through diet	0.03	21.97
Informing the patient that are overweight/obese	0.005	28.11
** Fasting blood glucose (R^2^ = 0.48) **		
Following the recommendation to reduce the consumption of sugars	0.01	12.44
Knowing the actual value of fasting blood glucose	<0.0001	26.05
Knowing the recommended value for fasting blood glucose	0.003	14
Informing the patient that they are overweight/obese	0.03	8.77
** Glycated hemoglobin—type 2 diabetes mellitus(R^2^ = 0.50) **		
Knowing the actual value of HbA1c	<0.0001	56.2
Knowing the recommended value for HbA1c	<0.0001	48.8
Informing and training diabetic patients regarding optimal blood sugar control	<0.0001	64.8

## Data Availability

The data and materials can be provided upon reasonable request to the corresponding author.
